# Ca^2+^ Ions Decrease Adhesion between Two (104) Calcite Surfaces as Probed by Atomic Force Microscopy

**DOI:** 10.1021/acsearthspacechem.1c00220

**Published:** 2021-10-04

**Authors:** Joanna Dziadkowiec, Matea Ban, Shaghayegh Javadi, Bjørn Jamtveit, Anja Røyne

**Affiliations:** † NJORD Centre, Department of Physics, University of Oslo, Oslo 0371, Norway; ‡ Materials Testing Institute, University of Stuttgart, Pfaffenwaldring 2b, 70569 Stuttgart, Germany

**Keywords:** calcite, adhesion, atomic force microscopy, surface forces, Ca^2+^ ions, mineral−fluid interactions

## Abstract

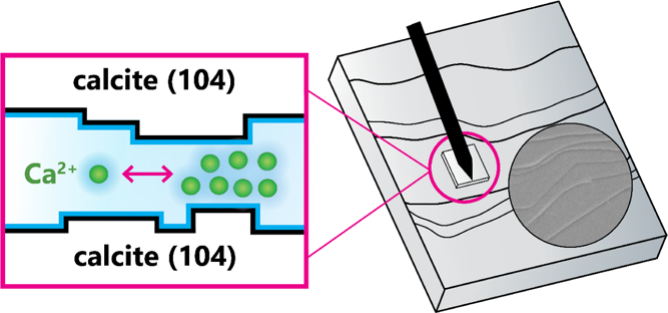

Solution composition-sensitive disjoining pressure acting between the mineral surfaces in fluid-filled granular rocks and materials controls their cohesion, facilitates the transport of dissolved species, and may sustain volume-expanding reactions leading to fracturing or pore sealing. Although calcite is one of the most abundant minerals in the Earth’s crust, there is still no complete understanding of how the most common inorganic ions affect the disjoining pressure (and thus the attractive or repulsive forces) operating between calcite surfaces. In this atomic force microscopy study, we measured adhesion acting between two cleaved (104) calcite surfaces in solutions containing low and high concentrations of Ca^2+^ ions. We detected only low adhesion between calcite surfaces, which was weakly modulated by the varying Ca^2+^ concentration. Our results show that the more hydrated calcium ions decrease the adhesion between calcite surfaces with respect to monovalent Na^+^ at a given ionic strength, and thus Ca^2+^ can sustain relatively thick water films between contacting calcite grains even at high overburden pressures. These findings suggest a possible loss of cohesion and continued progress of reaction-induced fracturing for weakly charged minerals in the presence of strongly hydrated ionic species.

## 1 Introduction

Calcite is one of the most ubiquitous nonsilicate, rock-forming minerals found abundantly in many distinct geological environments. Biogenic calcite builds extensive, often oil-bearing, limestone and chalk sedimentary deposits, while inorganic calcite precipitates as cement, mineral coatings, and vein-filling material in many types of sedimentary, metamorphic, and igneous rocks. Despite the richness of calcite’s depositional and growth environments, the most thermodynamically stable and abundant calcite face in most cases is the (104) cleavage plane. The interfacial properties and cohesion of (104) calcite surfaces in contact with geologically relevant solutions are suggested to influence several major deformation processes, including chemomechanical weathering,^1^ fluid-induced subsidence and water-weakening,^2−4^ subcritical fracturing,^5^ and carbonate-hosted seismicity.^6^ Solution composition-dependent disjoining pressures (DPs), associated with water films sustained on mineral grains in calcite-bearing rocks and in a wider range of mineralogical settings, may also control reaction-driven fracturing processes^7,8^ and damage by salt crystallization.^9^ More information is needed on the ion-specificity of surface forces and cohesion in all of these systems to understand the nanoscale details of these common deformation processes.

The forces acting between calcite surfaces in aqueous solutions are closely linked with the composition of the calcite–fluid interfaces, and there has been significant progress toward understanding the molecular details of these strongly hydrophilic^10^ interfaces. The hydrophilic nature of calcite surfaces has been attributed to the presence of surface-bonded hydrolysis H^+^ and OH^–^, species detected in air and water in surface-sensitive spectroscopic and diffraction experiments by Stipp and Hochella.^11^ This specific adsorption of water onto calcite bulk termination results from the presence of dangling bonds and under-coordinated surface Ca and O atoms.^12^ Later experimental and computational studies have provided evidence that the hydration layer detected on flat (104) calcite surfaces consists of associatively absorbed and highly ordered water molecules.^13−15^ Between two and five associatively adsorbed water layers, distinct from the bulk water, have been resolved on flat (104) calcite surfaces by various approaches, with differences mainly in the strength and type of bonding with the surface, location over surface >Ca or >CO_3_ groups, density and the extent of ordering, and surface residence times.^16−22^

Despite these different descriptions of the calcite hydration layer, there is a general agreement that inorganic ions do not bind directly to the calcite surface but reside on top of the surface-water layers as hydrated outer-sphere species.^16,23−27^ The location of the plane of outer-sphere complexation depends on the ion hydration shell properties, with Na^+^ ions bound more strongly and closer to the surface than more hydrated Ca^2+^ ions.^24,26^ Although Ca^2+^ is located further from the surface, it is, together with CO_3_^2–^ species, considered to be the main potential-determining ion for calcite.^28^ On the contrary, inert Na^+^ cations do not neutralize the surface charge of calcite even at high molar concentrations and affect the ζ-potential of calcite to a smaller extent than Ca^2+^ (as reported for Na^+^ concentrations varying between 0.05 and 2 M).^23,29^ The ion-specific location of the complexation plane observed for calcite complicates the definition of the electrical double-layer (EDL), and thus the interpretation of surface forces acting between calcite surfaces in aqueous solutions.

Since the measurement of forces between the two calcite surfaces is experimentally challenging, these forces have been directly measured and distance-resolved only in few works using atomic force microscopy (AFM) and a surface force apparatus (SFA).^30−34^ Repulsive or slightly adhesive forces act between the two calcite surfaces in calcite-saturated water and have been attributed to hydration repulsion that counteracts the attractive van der Waals (vdW) forces. Higher ionic strength (IS) due to the addition of NaCl or the presence of sulfate ions has been found to increase the adhesion, likely as an effect of EDL screening and attractive ion correlation forces. Reactivity, recrystallization, and the resulting surface roughening of calcite surfaces often correlate with a much higher magnitude and range of repulsion that would be expected from the theoretical EDL contribution.^33^

AFM force measurements in a dissimilar surface configuration with one calcite surface against a smooth and less-reactive surface (such as colloidal silica, gold, or substrates with modified hydrophilicity) are less experimentally challenging and provide a higher distance resolution.^35−38^ These works point to the dominant effect of ion-specific hydration on the repulsive forces measured at the smallest surface separations and show a significant decrease of the measured adhesion in the presence of Ca^2+^. The strong ion-specific effects on adhesion between calcite and a functionalized AFM tip have also been demonstrated in a molecular dynamics study for several different crystallographic planes of calcite.^39^ However, such measurements cannot be directly related to two interacting calcite surfaces because of the presence of another, chemically different interface. Thus, the complete understanding of the ion-specific forces, which govern the cohesion of two calcite grains in the presence of surface potential-determining or inert inorganic electrolyte ions, is still lacking.

In this paper, we present AFM measurements of forces acting between two cleaved (104) calcite surfaces in aqueous solutions containing low or high concentrations of Ca^2+^, which is a calcite surface potential-determining cation. We show the dependence of the measured adhesion on the Ca^2+^ concentration and discuss the origin of adhesive forces in relation to hydration forces, the streaming potential of calcite, van der Waals attraction, and ion correlation. Our work contributes to the systematic understanding of adhesion between the two calcite surfaces in the presence of potential-determining or inert ions with implications for rock deformation processes.

## 2 Materials and Methods

### 2.1 AFM Experimental Setup

**Figure 1:**
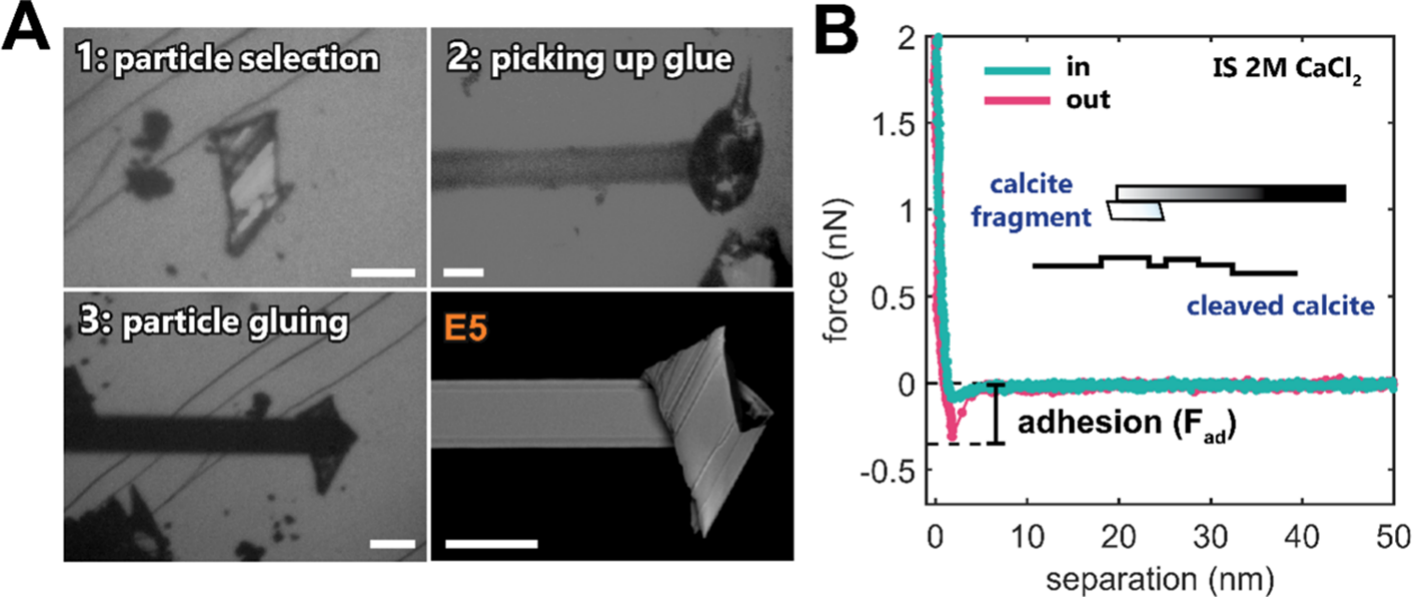
AFM experimental setup. (A) Preparation of a calcite-modified AFM probe: (1) a freshly cleaved (104) calcite crystal surface is scanned for small calcite fragments, which rest on the exposed surface after cleaving the crystal. A suitable, flat, relatively smooth, and ∼50 μm large calcite particle is selected; (2) a tiny droplet of epoxy glue is picked up with a tipless AFM cantilever by touching the glue droplet at a very small applied load (∼1 nN); and (3) the glue-covered cantilever is moved back to the location of the preselected calcite fragment and lowered onto the particle at a higher load (∼10 nN). The glue is cured overnight at room temperature, with the cantilever constantly pressing onto the calcite fragment. The images 1–3 are from an optical microscope. The scanning electron microscopy (SEM) image of the calcite-modified cantilever is shown in the bottom right (used in experiment E5, see Figure 2). The scale bar in each panel corresponds to 50 μm. (B) Representative force curve measured as a function of a separation between the two calcite surfaces (a calcite-modified probe and a freshly cleaved (104) calcite surface) in CaCl_2_ solution (ionic strength, IS, of 2 M; presaturated with respect to calcite). Adhesion (*F*_ad_) is determined as the absolute value of the negative attractive force (pull-off force) measured on retraction.

The forces between the two freshly cleaved (104) calcite surfaces were measured using a JPK NanoWizard AFM (equipped with an Olympus IX71 microscope) in a force spectroscopy mode. We used high purity and high-quality Iceland Spar calcite and cleaved all of the samples from the same larger single calcite crystal. The design of the AFM liquid cell with a ∼4 mL volume and the preparation of calcite-modified AFM cantilevers were both adapted with no changes from Javadi and Røyne.^31^ Only the relatively smooth calcite surfaces with no evident step edges over large areas were chosen for the measurements, as observed in the top and bottom view AFM optical microscope with the resolution of 0.35 and 0.22 μm/pixel, respectively. As the cantilever width is smaller than the sizes of the chosen calcite particles, we can verify that a calcite particle remains glued to the cantilever at all times (Figure 1A). The sensitivity of each tipless cantilever (All In One-TL, 15 kHz, 0.2 N/m) before modification was measured using a thermal tune calibration method. The sensitivities of calcite-modified cantilevers were extracted from the contact-based force–distance curves. The raw AFM data were processed using JPKSPM and MATLAB software. Adhesion (*F*_ad_) is determined as the absolute value of the negative attractive force (pull-off force) measured on retraction (Figure 1B). Significant adhesive peaks are chosen with *F*_ad_ larger than the value of 3 standard deviations of the noise level extracted from the force–distance curve at higher separations (>10^–2^ nN). The cantilever movement velocity was set to below 200 nm/s to ensure no observable hydrodynamic effects in the measured force–distance curves. The applied normal load (setpoint) was, in all cases, below 50 nN. The temperature in the isolated AFM enclosure never changed by more than 0.5 °C over the whole duration of each experiment (∼8 h). In all experiments, we always measured the forces in two contact positions on the bottom cleaved calcite surface to ensure a good experimental reproducibility. We always reported pull-off adhesion forces (*F*_ad_; Figure 1B) with no distinction for these two contact positions, as we rarely observed a major difference between the *F*_ad_ measured in two locations. For each solution and contact position, we measured hundreds of individual force–distance curves. The pH of the used solutions was frequently measured in a liquid cell replica (with a similarly sized freshly cleaved calcite fragment) placed inside the AFM chamber (due to the lack of space in the actual liquid cell).

### 2.2 Solutions

**Figure 2:**
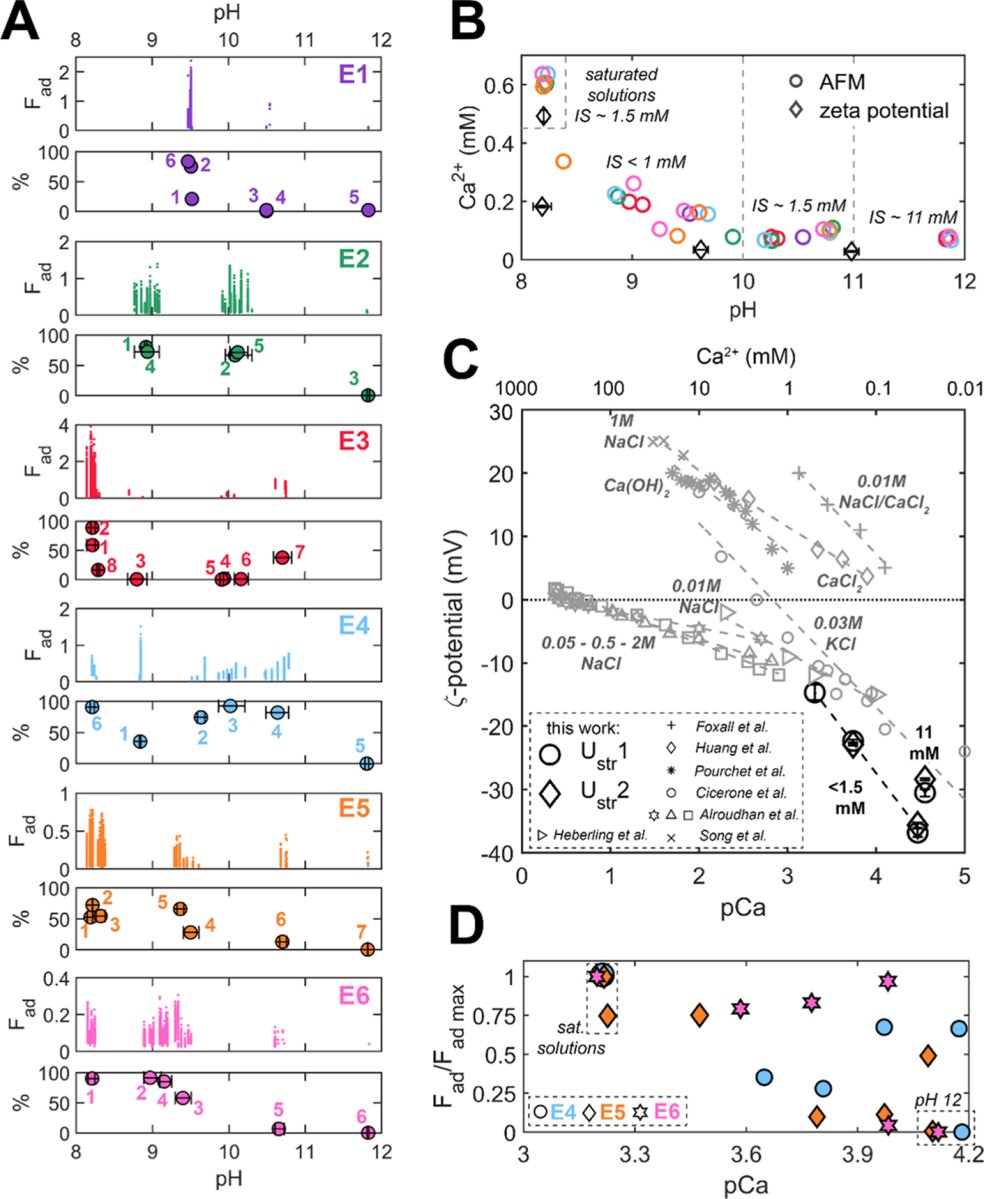
Low ionic strength experiments in water presaturated with calcite and pH adjusted with NaOH (type A solutions): (A) adhesion measured between the two calcite surfaces as a function of pH. The panels show adhesion (pull-off force; *F*_ad_ in nN; note the varying scales on *y*-axes) for each measured adhesive force–distance curve and the corresponding percentage of adhesive force–distance curves for each solution (%) in experiments E1–E6, each with a different pair of calcite surfaces. The numbers correspond to the solution injection order. More than 200 individual force–distance curves were measured for each injected solution. (B) Concentration of Ca^2+^ in the type A solutions with indicated ionic strength (IS); (C) ζ-potential (ζ) of calcite as a function of Ca^2+^ concentration (pCa = −log_10_(Ca^2+^ [M])) in solutions with the ionic strength <1.5 mM for pH 8, 9, and 10 solutions and IS = 11 mM for pH 11 solution. *U*_str_ is the streaming potential measured in duplicates (*U*_str_1, *U*_str_2). Data from the literature^16,29,30,50−53^ is shown in gray for comparison (natural limestone reported by Alroudhan et al.^29^ with IS of 0.05 M—squares, 0.5 M—triangles and 2 M—stars, ground Iceland spar reported by Heberling et al.,^16^ and synthetic granular calcite in the other works). The dashed lines connect data measured at equal ionic strength indicated along with the background electrolyte (IS reported by Huang et al.^51^ and Pourchet et al.^30^ was not additionally adjusted); (D) average adhesion measured in E4–E6 plotted as a function of Ca^2+^ concentration. Each adhesion value was normalized with respect to the maximum *F*_ad_ measured in a given experiment in the water fully saturated with CaCO_3_ (pH 8.3).

We measured forces between two calcite surfaces in two types of solutions. The first set of experiments (type A solutions) was performed in low Ca^2+^ concentration solutions with varying pH and no CaCl_2_ added (experiments E1–E6): Here, we first used NaOH to adjust the pH of the water to 10, 11, or 12 and subsequently added calcite powder (∼1 g/L; Merck Supelco) to saturate the solutions with calcite. The solutions were gently stirred for 1 day in open 1 L volumetric flasks. We then stopped CO_2_ dissolution into the solutions (before the solutions became saturated with the dissolved CO_2_ at the atmospheric pCO_2_ = 10^–3.5^ atm) by closing and sealing the flasks tightly with parafilm. As such, we obtained solutions with a varying amount of dissolved Ca^2+^ and pH higher than 8.3 (before the solutions reached the full saturation with respect to calcite at atmospheric pCO_2_). The most basic solutions (initial pH 12) had the lowest amount of dissolved Ca^2+^. We additionally prepared Milli-Q water solutions that were fully saturated with calcite and atmospheric CO_2_, in which no NaOH was added and the solutions were vigorously stirred with calcite powder in open volumetric flasks until they reached a pH value of 8.2–8.3. The dissolved concentration of Ca^2+^ was always measured for each used solution collected immediately before the solution injection into the AFM liquid cell (using the Dionex ICS-1000 Ion Chromatography System). The pH of the solutions was monitored throughout each experiment. With these two parameters, the full solution speciation could then be calculated using PhreeqC software.^40^Figure 2B shows Ca^2+^ concentration plotted as a function of pH for all used type A solutions.

The second set of experiments was performed in calcite presaturated CaCl_2_ solutions with ionic strength (IS) between 0.25 and 2 M (E7–E14; type B solutions). CaCl_2_ solutions were vigorously stirred with the added calcite powder (∼1 g/L) in open volumetric flasks for about 10 days until they reached a full equilibrium with calcite at atmospheric pCO_2_ conditions. All solutions were filtered with 0.2 μm polyether–sulfone syringe filters before the injection into the AFM liquid cell. We also analyzed the dissolved Ca^2+^ concentration in some of the solutions collected from the AFM liquid cell after the experiments; however, no major, reproducible changes in comparison with the initial Ca^2+^ concentrations were detected. The detailed solution parameters are given in Tables S1 and S2 in the Supporting Information (SI).

### 2.3 ζ-Potential

ζ-Potential (ζ) of the (104) Iceland spar calcite surface was determined by the streaming potential method with the SurPASS Electrokinetic Analyzer (Anton Paar, Austria) in low Ca^2+^ concentration calcite-saturated solutions prepared as for the AFM experiments. In this setup, the potential difference is generated by the movement of a liquid relative to a flat macroscopic calcite crystal, and the streaming potential-derived ζ was calculated as follows:
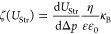
1where d*U*_Str_/dΔ*p* represents the streaming potential coupling coefficient [V·Pa^–1^], η is the dynamic viscosity of the electrolyte [Pa·s], ε is the electrolyte dielectric constant, ε_o_ is the permittivity in a vacuum [F·m^–1^], and κ_B_ is the conductivity of the bulk electrolyte solution [S·m^–1^]. We performed the measurements in a clamping cell attachment to facilitate the use of a large and brittle calcite (104) surface (which is additionally less reactive than small, micron-sized crushed aggregates), following the methodology by Ban et al.^41^ The microchannel’s walls are made of the reference polypropylene (PP) surface, which opposes the studied calcite surface. In this asymmetrical surface configuration, the surface charge of PP affects the overall measured ζ, by making it more negative (the fractional contribution of PP to the overall determined ζ can be up to 0.5; however, the higher surface area due to calcite roughness generally lowers the contribution of PP to the overall ζ in an asymmetric surface configuration).^41^ The ζ-potential of PP (with pZc = 4) is around −80 mV at high pH (8–9).^42^ The measurements were performed in duplicates for each tested solution, and each data point in Figure 2C corresponds to 12 measurements of ζ acquired within 45 min. Prior to testing, the cleaved calcite’s surface was cleaned with pressurized argon to remove loose dust and rinsed in the probed electrolyte solution for approx. 5 min to assure electrode conditioning, laminar flow conditions, and proper wetting of the surface. Between each used solution, we cleaned the calcite sample by flowing Milli-Q water over the surface for approx. 5 min and the instrument was cleaned by measuring the conductivity of the wastewater until it reached the Milli-Q water conductivity. Before and after each test cycle, a solution sample was collected and Ca^2+^ concentration was analyzed with inductively coupled plasma mass spectrometry (ICP-MS; iCAP-TQ, Thermo; samples digested in 2% HNO_3_). The recorded parameters of streaming potential permit the analysis of ζ only in low and medium ionic strength solutions as those used in experiments E1–E6. Solution parameters are shown in Figure 2B and Table S3.

### 2.4 Force Modeling

The Derjaguin–Landau–Verwey–Overbeek (DLVO) disjoining pressure (*P*_DLVO_) acting between two flat calcite surfaces was calculated as the sum of van der Waals attraction (*P*_vdW_) and electrical double-layer repulsion (*P*_EDL_), using the equations given in Israelachvili (2011)^43^ (pages 255, 316 therein)
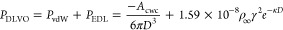
2
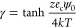
3
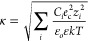
4where *A*_cwc_ is the Hamaker constant for two calcite surfaces across the water, *D* is the separation between the surfaces (m), ρ_∞_ is the bulk electrolyte concentration (M), 1/κ corresponds to the Debye length (1/*m*), *z* is the ion valency, *e*_c_ is the elementary electric charge (C), ψ_0_ is the surface potential (here estimated as the ζ-potential at the slipping plane; V), *k* is the Boltzmann constant, *T* is the temperature (K), *C*_*i*_ is the concentration of each ionic species *i* in the bulk solution (M), ε_0_ is the electrical permittivity of vacuum (F/m), and ε is the dielectric constant of pure water. The concentrations of ionic species in the solutions were modeled using PhreeqC software^40^ based on the measured Ca^2+^ concentration, and pH values and all of the present ionic species have been included in the Debye length calculation according to eq 4.

The repulsive pressure due to surface roughness (*P*_rough_) was estimated by fitting a simple exponential term^44^
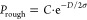
5where *C* is the fitting constant (Pa) and σ is the root-mean-square (rms) surface roughness (m).

The probability of strong ion correlation was estimated using the ionic fluctuation parameter ∑^45,46^
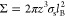
6
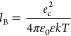
7where σ_s_ is the surface charge density of a planar surface (number of charges/m^2^) and *l*_B_ is the Bjerrum length (m).

## 3 Results

Figure 1B shows representative force–distance curves measured between the two cleaved calcite surfaces using a calcite-modified AFM probe, as shown in panel A. Throughout this work, we focus on adhesion values (pull-off force measured on retraction; see Figure 1B). Although our symmetric system with two calcite surfaces is experimentally challenging due to undefined contact topography, our work provides strong insight into ion-dependent adhesion. The measured adhesion depends on the “real” contact area between the two cleaved calcite surfaces. Because of the cleaved calcite’s undefined and stepped topography, we expect the real contact area and thus the measured adhesion to vary significantly for various pairs of calcite surfaces, mainly because of the micron-scale defects in the surface topography, such as step edges that define the actual contact area. Nevertheless, instead of comparing the absolute adhesion values across experiments, we follow changes in adhesion in a given experiment for a fixed contact region, and we focus on how the adhesion responds to changes in solution chemistry. Such response of adhesion to the changing solution chemistry provides robust characterization of solution composition-dependent calcite cohesion in a symmetrical surface configuration: the confined ionic species determine how close the two surfaces can approach each other, which influences the measured adhesive forces.^47−49^ We record hundreds of force curves for each solution condition and inject solutions in changeable order.

### 3.1 Low Ca^2+^ Concentration Calcite-Saturated Water

Figure 2A compares the adhesion measured in low Ca^2+^ concentration type A solutions, presaturated with respect to calcite in six experiments, each with a different pair of calcite surfaces. The pH of the solutions was adjusted between 9 and 12 to obtain saturated solutions with varying concentrations of dissolved Ca^2+^. In experiments E3–E6, we additionally used “fully” calcite-saturated solutions, in equilibrium with the atmospheric pCO_2_, with no added NaOH, and pH stabilized at 8.3. The Ca^2+^ concentration and pH of all type A solutions used for experiments in Figure 2A are plotted in Figure 2B.

The forces measured for pH 12 type A solutions (lowest Ca^2+^ concentration) were always repulsive, with near 0% adhesive force runs in all six experiments. Adhesion values (pull-off forces; *F*_ad_) and the percentage of adhesive force runs for type A solutions with pH ranging from 8.3 to 11 were both variable, but generally, these two parameters were the highest for the fully saturated type A solutions (pH 8.3) and decreased with increasing pH and lower concentration of dissolved Ca^2+^ in the solutions.

The ζ-potential measurements in calcite presaturated water with pH ranging from 8.3 to 11 shown in Figure 2C indicate that the ζ-potential became less negative with increasing concentration of dissolved Ca^2+^. The least negative ζ-potential was measured for the saturated solution in equilibrium with atmospheric pCO_2_ and pH 8.3 (we plot the ζ-potential as a function of pCa^2+^ since H^+^ and OH^–^ are not potential-determining ions for calcite^23,50^). The same trend has been generally found in many other works, in spite of using various types of calcite grains and methodologies differing from ours, as compiled in Figure 2C.

In Figure 2D, the average adhesion measured in experiments E3–E6 is additionally expressed as a function of Ca^2+^ concentration in the used solutions (nonadhesive forces were included as *F*_ad_ = 0) and normalized with respect to the highest average adhesion measured in the given experiment (which in all cases was the value for fully saturated solutions at pH 8.3). Again, the saturated solutions with the highest concentration of Ca^2+^ generally showed the highest adhesion, all solutions (pH 12) with little dissolved Ca^2+^ (and 10 times higher IS due to NaOH addition) exhibited repulsion, and solutions with pH 9 to 11 showed a quite variable adhesion.

### 3.2 High Ca^2+^ Concentration Calcite-Saturated CaCl_2_ Solutions

**Figure 3:**
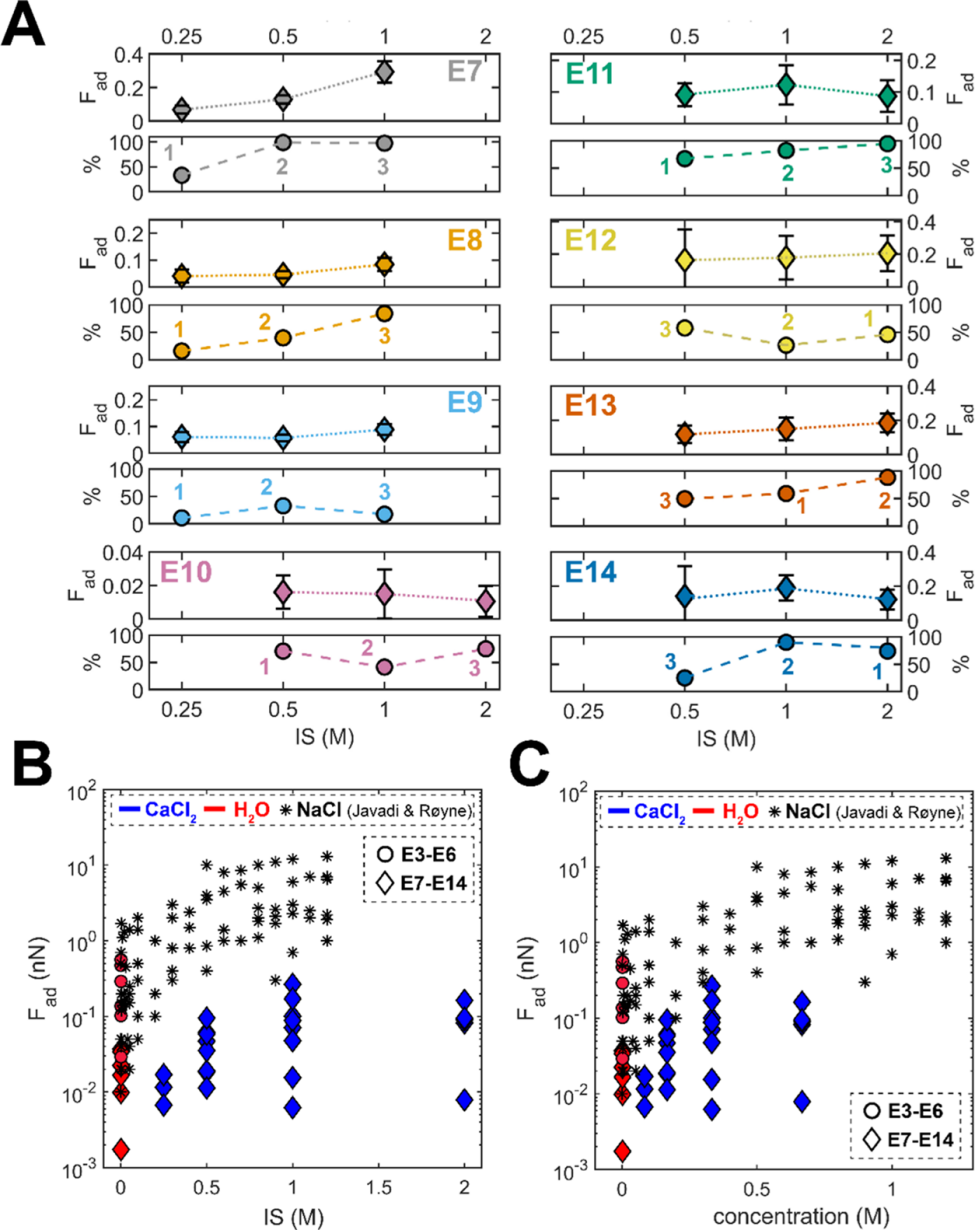
High ionic strength experiments in type B calcite-saturated CaCl_2_ solutions: (A) adhesion measured as a function of solution ionic strength (IS). The panels show the average adhesion (pull-off force; *F*_ad_ in nN; note the varying scales on *y*-axes) measured for all adhesive force–distance curves for each type B solution and the corresponding percentage of the adhesive force–distance curves (%) in experiments E7–E14, each with a different pair of calcite surfaces. Each data point corresponds to more than 200 measured individual force–distance curves. The numbers correspond to the solution injection order; (B) average adhesion measured in all experiments as a function of solution ionic strength (IS). Here, the nonadhesive force curves were also included in the average calculation as *F*_ad_ = 0. Blue data corresponds to data points measured in CaCl_2_ solutions and red points show the measurements in calcite-saturated water (H_2_O; only data for the fully saturated solutions with pH 8.3 are plotted). Diamonds show the data from E7–E14 experiments (shown in A, all type B solutions) and circles correspond to E3–E6 experiments (type A pH 8.3 solutions); Figure 2A, only pH 8.3. Adhesion measured in the same setup by Javadi and Røyne (2018)^31^ in NaCl solutions saturated with calcite is plotted with black asterisks; (C) data from panel (B) expressed as a function of electrolyte concentration.

Figure 3A compares adhesion measured in the high concentration, calcite-saturated CaCl_2_ type B solutions with IS between 0.25 and 2 M. In most of the experiments, the measured adhesion and the percent of adhesive force runs increased with the increasing CaCl_2_/CaCO_3_ ionic strength. However, the measured adhesion was, in general, very low in all experiments (below 0.4 nN). The increase in adhesion with the increasing solution IS was not an artifact of the solution injection order (in most experiments, the solutions were injected in the order of increasing IS), as verified in experiments E12 to E14, with an altered injection order.

**Figure 4:**
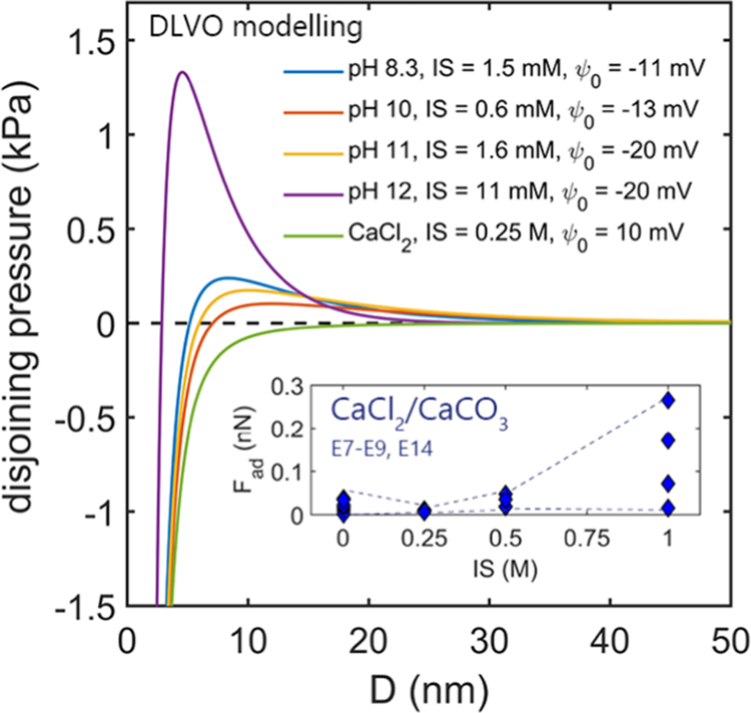
Modeling of the DLVO disjoining pressure as a function of separation (*D*) between the two flat calcite surfaces in low concentration type A solutions with pH between 8.3 and 12 and lowest IS = 0.25 M type B CaCl_2_/CaCO_3_ solution (see eqs 2–4). The indicated surface potential values were adapted from Heberling et al.^16^ The Hamaker constant for two calcite surfaces across the water *A*_cwc_ = 1.44 × 10^–20^ J was adapted from Bergström.^58^ The IS strength of the solutions was calculated using PhreeQc software^40^ and verified across the measured pH and Ca^2+^ concentrations. In all CaCl_2_/CaCO_3_ type B solutions, the EDL barrier collapses due to the high ionic strength of the solutions (only the IS = 0.25 M is shown). The inset shows the adhesion (*F*_ad_) measured in AFM experiments (E7–E9 and E14, see Figure 3A), in which both calcite-saturated solution with no added CaCl_2_ (CaCO_3_) and calcite-saturated CaCl_2_ solutions (CaCl_2_/CaCO_3_) with ionic strength (IS) between 0.25 and 1 M were used for the same set of calcite surfaces (the dashed lines are to guide the eye).

The average adhesion measured in experiments E7–E14 (type B solutions), as well as the adhesion measured in CaCO_3_-saturated water (only at pH 8.3) in experiments E3–E6, is summarized as a function of ionic strength in Figure 3B, and as a function of electrolyte concentration in Figure 3C. Note that in some high concentration experiments, we additionally measured adhesion in pH 8.3 CaCO_3_-saturated water (red diamonds; E7–E9, E14; also shown in the inset of Figure 4). We further compared our data in CaCl_2_/CaCO_3_ solutions with the measurements in NaCl/CaCO_3_ solutions reported by Javadi and Røyne,^31^ who used an identical AFM setup. While the magnitude of the adhesive forces measured in calcite-saturated water was comparable in both works, the adhesion we measured in CaCl_2_/CaCO_3_ was even 3 orders of magnitude lower than that reported for NaCl/CaCO_3_ solutions at a given IS or concentration (the reported Ca^2+^ content in NaCl/CaCO_3_ solutions was below 1.3 mM at all used NaCl concentrations, being significantly lower than the Ca^2+^ concentration in our CaCl_2_/CaCO_3_ solutions^31^). Our control measurements in pH 8.3 solutions in some of the high concentration experiments (red diamonds; E7–E9, E14) generally showed lower adhesion than in E3–E6 experiments (red circles). Despite this, we never measured any high pull-off forces that would be comparable in magnitude to the NaCl/CaCO_3_ data (apart from calcite-saturated water) in any of the eight experiments (E7–E14). This indicates that a significant concentration of Ca^2+^ lowers the adhesion between the two calcite surfaces with respect to Na^+^. In addition, we observed that the adhesion measured in the 0.25 M IS CaCl_2_/CaCO_3_ solution was reproducibly lower than in calcite-saturated water at pH 8.3 for given pairs of calcite surfaces (E7–E9; the inset in Figure 4). Such minimum in adhesion was not detected in NaCl/CaCO_3_ solutions.

## 4 Discussion

### 4.1 Repulsive Force Contributions

Flat (104) calcite surfaces exhibit a rather low surface charge^54,55^ (of −0.02 up to ∼−0.1 C/m^2^). This low surface charge of calcite limits the height of possible EDL repulsive barrier to several kPa (as estimated assuming the limiting surface potential of −30 mV). However, the magnitude of EDL repulsion depends not only on the surface charge but also on the ionic strength of the solution and the ability of a given ionic species to screen the mineral’s surface charge. The main potential-determining ions for calcite surfaces are not H^+^ and OH^–^ but Ca^2+^ and CO_3_^2–^.^23,50^ As such, we expect that the concentration of Ca^2+^ has a considerable effect on the magnitude and range of EDL repulsive forces and thus on the measured adhesion.

Indeed, our and literature^16,29,30,50−53^ ζ-potential data compared in Figure 2C indicate that even small variations in the Ca^2+^ concentration can significantly alter the ζ-potential of calcite, with the extrapolated point of zero charge (pZc) varying between 0.1 and 10 mM of dissolved Ca^2+^ for various types of calcite surfaces (and a much larger Ca^2+^ needed to reach the pZc for limestone rocks). In contrast, the nonpotential determining Na^+^ ions have a much weaker influence on the measured ζ-potential (as shown for Na^+^ concentrations varying between 0.05 and 2 M in Figure 2C) as Na^+^ does not significantly affect the surface charge of calcite even at high molar concentrations.^29^

Using the ζ-potential values, we modeled the total DLVO contributions (EDL + van der Waals; vdW; eqs 2–4) for low Ca^2+^ concentration calcite-saturated water with pH from 8.3 to 12 (type A solutions) and for the lowest IS CaCl_2_/CaCO_3_ type B solution, as plotted in Figure 4. To estimate the EDL repulsion between the two calcite surfaces, we used the experimental streaming potential-derived ζ-potential values reported by Heberling et al.^16^ for crushed Iceland spar calcite, which are the closest to our experimental conditions (we interpolated the ζ-potential from the measurements shown in Figure 7 in Heberling et al.,^16^ at pH values corresponding to our solutions). This is because our streaming potential data is to some extent more negative due to the presence of the polypropylene reference surface (see 2Section 2) (using our ζ-potential values would yield a higher magnitude of the modeled EDL repulsion; however, the overall trend would remain similar; please refer to Figure S1 in the SI).

The highest repulsive EDL barrier exists for the pH 12 calcite-saturated water. We attribute this barrier to the very low concentration of dissolved Ca^2+^ (<0.1 mM; Figure 2B) and a considerable amount of Na^+^ (IS ∼11 mM). The lack of Ca^2+^ prevents screening of the calcite surface charge and leads to a substantial accumulation of inert Na^+^ close to calcite surfaces. As such, Ca^2+^-free, high pH solutions (calcite dissolution is also suppressed at such high pH) exhibit repulsion between calcite surfaces and should have a stabilizing effect on calcite suspensions. This is in very good agreement with our AFM measurements, in which no adhesion was reproducibly measured for all pH 12 solutions in all experiments (E1 and E2, E4–E6; Figure 2A).

The DLVO disjoining pressure modeled for calcite-saturated water with pH ranging from 8.3 (fully saturated) to pH 11 (less dissolved Ca^2+^) displays much lower heights of the repulsive EDL barrier in comparison with pH 12 solution (Figure 4). The EDL heights are comparable, with shorter Debye lengths for the fully saturated solution at pH 8.3. For our terraced and stepped calcite surfaces, these small differences in the EDL range determine how many adhesive contacts can be established during force measurements, leading to small but reproducible differences in adhesion. The adhesion and the percent of adhesive force runs (Figure 2A) were slightly higher for the fully saturated pH 8.3 solution (the lowest negative ζ-potential and the shorter EDL range), and lower but more variable adhesion was measured for the pH 9–11 solutions (comparable EDL magnitude but larger Debye length). As such, our data in low IS solutions show that very low mM concentrations of potential-determining Ca^2+^ were likely sufficient to screen the electrostatic repulsion between calcite surfaces and yield adhesion.

Our DLVO modeling in Figure 4 further shows that for high IS CaCl_2_/CaCO_3_ solutions, in the presence of a high concentration of Ca^2+^, the electrostatic EDL barrier fully collapses, leaving attractive vdW forces to dominate at all separations (as plotted for 0.25 M IS type B CaCl_2_/CaCO_3_ solution). Based on this simple DLVO model, we would expect to measure much higher adhesion than for the low Ca^2+^ concentration solutions, where significant electrostatic repulsion is present. However, we only measured very weak adhesion at high Ca^2+^ concentrations in all experiments. As we use calcite-saturated solutions in equilibrium with atmospheric pCO_2_, we do not observe and rule out any significant micron-scale surface roughening of the chosen calcite contact in the timescale of our experiments. This is in agreement with the results shown in Javadi (2019; pages 111–117 therein),^56^ who demonstrated slight smoothing of the contact area between the two cleaved calcite surfaces during repeated force measurements in an identical AFM setup. As such, our results likely indicate the presence of non-DLVO repulsive force components that prevent the calcite surfaces from forming strong adhesive contacts despite the expected EDL collapse. We attribute this additional repulsion to the hydration of the outer-sphere adsorbed Ca^2+^ species. Accumulation of hydrated Ca^2+^ in heavily collapsed EDLs can lower the adhesion, as there is a considerable energy penalty required to squeeze these hydrated counterions out of the interfacial region.^35^

### 4.2 Attractive Force Contributions

In most solution conditions, apart from pH 12 solutions, we measured small adhesion between the two calcite surfaces (Figures 2 and 3). Adhesion between like-charged solid surfaces generally originates from the attractive van der Waals forces. The adhesion that we measured was generally weak because of the cleaved calcite’s stepped topography. The roughness of a step-free cleaved (104) Iceland spar calcite surface is extremely low (∼1 Å^57^), but in our case, undefined terraced topography will drastically limit the real contact areas.

**Figure 5:**
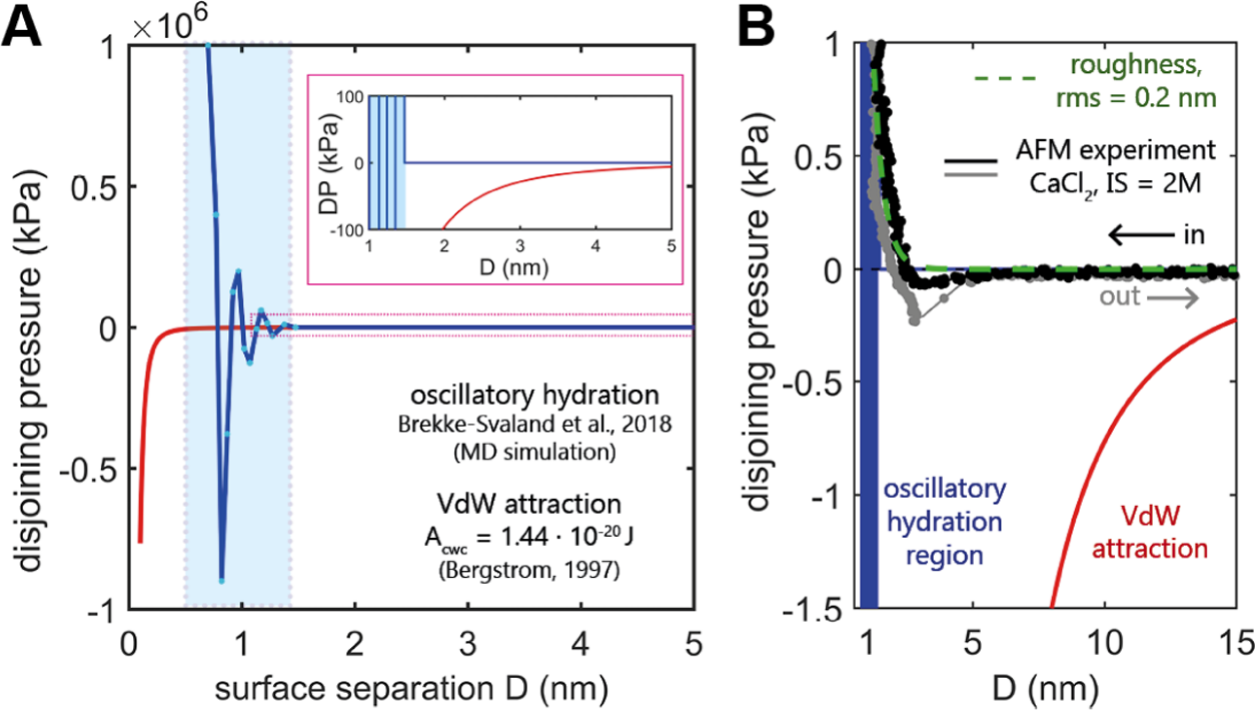
(A) Possible negative contributions (attractive forces) to the disjoining pressure acting between the two calcite surfaces: the oscillatory hydration force with attractive and repulsive minima adapted from the molecular dynamics simulation between ideally flat (104) calcite surfaces as reported by Brekke-Svaland and Bresme^60^ and attractive van der Waals forces (vdW) calculated for two calcite surfaces across water (Hamaker constant *A*_cwc_ = 1.44 × 10^–20^ J, ref (58)). The inset zooms on the disjoining pressure (DP) region between −100 and 100 kPa. (B) Experimental AFM data (E11) plotted onto the theoretical contributions shown in subplot A. The measured forces were expressed as pressure assuming that the real contact area between the two calcite surfaces was 0.5% of the total area of the calcite particle attached to an AFM tip. The roughness contribution was estimated assuming the calcite’s root-mean-square (rms) value of 0.2 nm (eq 5).

Nevertheless, we can estimate the expected range of vdW forces using the Hamaker constants reported in the literature and comparing adhesion values to other roughness-free surfaces, such as mica. Adhesion between the two calcite surfaces in water should be only 35% lower than for two micas (using the Hamaker constants across the water of 1.44 × 10^–20^ J for calcites^58^ and 2.2 × 10^–20^ J for micas^59^). The average adhesion measured with the SFA for atomically smooth mica surfaces in water can reach 63.3 ± 26.2 mN/m,^48^ which amounts to ∼160 kPa (with a contact area radius of ∼50 μm). The highest adhesion measured between calcite surfaces in water in this work was ∼4 nN. We assume that the real contact area between the two calcite particles in our study is only a very small fraction of the total tip-attached particle due to terraced and stepped calcite topography on both sides of the contact. With the real contact area being, for example, only 0.5% of the tip-attached calcite particle (which appears to be a reasonable value not to underestimate the measured adhesive disjoining pressure), the adhesive disjoining pressure in water is 40 times lower for calcite surfaces (∼4 kPa) than for micas (∼160 kPa). To reach the adhesion ratio determined from the Hamaker constants, the real contact area would have to be as low as 0.02% of the tip-attached calcite particle. Thus, it is more likely that the adhesion due to vdW is rather weakened by the presence of the hydration barrier, which prevents the surfaces from reaching the smallest surface separations where the separation distance-dependent vdW attraction increases rapidly with the decreasing surface separation (Figure 5).

It is generally understood that the hydration of calcite surfaces will weaken the cohesion between the surfaces, as it is not possible to squeeze out these strongly bound water molecules at moderate applied pressures. The recent molecular dynamics descriptions of the hydration layers on atomically smooth calcite^60,61^ display extremely strong repulsive and attractive disjoining pressure minima linked to the progressive confining and squeezing out of the ordered water molecules (Figure 4). Figure 5A shows vdW and oscillatory hydration for ideally smooth calcite surfaces in ion-free water, and the latter adapted from Brekke-Svaland and Bresme.^60^ Despite the existence of attractive minima, the first, smallest repulsive barrier is estimated to have a magnitude on the order of 10 MPa, making it effectively inaccessible for our range of applied loads to reach any of the attractive minima (assuming that oscillatory hydration interaction potential is valid at an asperity scale despite our surface roughness). In Figure 5B, we compare our experimental data with the theoretical vdW and hydration disjoining pressures. Assuming that the real contact area between the two calcite surfaces in our experiments is again only 0.5% of the total area of the calcite particle attached to the AFM tip, we estimated the pressure acting between our calcite surfaces to be on the order of a few kPa at the applied load that we used. As such, we again argue that calcite surfaces in our experiments are unlikely to experience any of the attractive hydration force minima, simply because the disjoining pressure required to remove even the outermost water layers (∼10 MPa) is too high. Instead, the weak adhesion is more likely to result from vdW forces: significant kPa attractive vdW disjoining pressure acts already at surface separations >15 nm, as shown in Figure 5.

### 4.3 Ca^2+^ Effects

The adhesion we measured in type B CaCl_2_/CaCO_3_ solutions was Ca^2+^ concentration-dependent and increased slightly at higher IS. Javadi and Røyne^31^ have reported a much more pronounced increase in adhesion between the two calcite surfaces with increasing NaCl/CaCO_3_ concentration in the identical AFM setup (Figure 3B,C) and attributed it partly to the growing importance of ion correlation effects. Following the discussion by Liberto et al.^46^ and using the equations from Netz and Orland,^45^ we can estimate the probability for the ion correlation to affect the forces between the two cleaved calcite surfaces by calculating the ionic fluctuation parameter ∑ (eqs 6 and 7). The larger the ∑, the more relevant is the ionic correlation and the resultant attraction of the EDLs,^62^ with ∑ < 1 generally indicating a negligible effect. Using the surface charge density of flat (104) Iceland spar calcite of 0.12 e^–^/nm^2^ reported by Lee et al.^55^ and in the presence of divalent Ca^2+^, we obtain ∑ ∼3. In comparison, ∑ drops to ∼0.4 for monovalent Na^+^. As such, the ion correlation effects should be much more pronounced in the presence of calcium and enhance the adhesion between calcite surfaces with respect to Na^+^. We did not observe such an increase, with adhesion in CaCl_2_ solution being even 2 orders of magnitude lower than that reported for NaCl solutions, making ion correlation effects due to localized multivalent ions such as Ca^2+^ not significant to calcite cohesion in our system in agreement with other works.^30,46^

The adhesion measured in NaCl/CaCO_3_ is generally up to 2 orders of magnitude higher^31^ than in our CaCl_2_/CaCO_3_ solutions, and we suggest that it is not likely to be caused by ion correlation interactions. Instead, the significant differences in adhesion likely arise because of the differences in adsorption of these two ions. Less hydrated Na^+^ ions (hydrated radius of 3.48 Å)^63^ can partially break the structure of strongly ordered surface-water molecules and bind much closer to the calcite’s surface than Ca^2+^, which is larger and more hydrated^24,26^ (hydrated radius of 4.12 Å).^63^ This allows the calcite surfaces to approach closer to each other and to be trapped in a deeper vdW minimum than in the presence of Ca^2+^. Thus, despite the Ca^2+^ cations being more likely to facilitate attraction due to ion correlations, their residence further away from the surface and significant hydration weaken the adhesion between calcite surfaces. Such cation-specific effects on ion adsorption and adhesion between surfaces are in agreement with the molecular modeling simulations reported by Jia et al.^64^ Weakly charged surfaces, such as calcite, can promote adsorption of less hydrated cations such as Na^+^, whereas the surface charge is insufficient to facilitate dehydration and transition to inner-sphere species for larger, more hydrated cations such as Ca^2+^. Conversely, strongly charged surfaces are more likely to drive the adsorption of more hydrated ions as the high surface charge density is sufficient to favor dehydration and binding of ions as inner-species over their interaction with water molecules in hydration shells. This is also in agreement with the repulsive forces observed for calcite in the presence of hydrated Mg^2+^ ions^36^ and significantly weaker adhesion reported for different sites on a calcite surface in the presence of Ca^2+^ with respect to Ca^2+^-free aqueous solutions in AFM measurements against a modified AFM tip.^37,39,65^

Although the adhesion is generally low at high Ca^2+^ concentrations (IS 0.5–2 M) in type B solutions, we observe a small but reproducible increase in the measured adhesion with increasing IS. This trend can be explained in two possible ways. Because of surface crowding, it should be easier to squeeze the hydrated Ca^2+^ out of the gap between the calcite surfaces as the electrostatic attraction for a given individual ion is now effectively weaker. Although Diao and Espinosa-Marzal^35^ have reported an increase of the energy needed to displace the hydrated Ca^2+^ at 100 mM concentration relative to 10 mM, even higher Ca^2+^ concentrations, corresponding to those used in this study, were not tested. Alternatively, the presence of Cl^–^ counterions, which can now enter near-surface regions in larger amounts, can weaken the secondary hydration barrier imposed by the outer-sphere Ca^2+^. A strong effect of anions on attractive and hydration forces between the calcite and gold-coated silica sphere has been reported by Guo and Kovscek.^36^

### 4.4 Implications

We showed that calcite cohesion in water and Ca^2+^-bearing solutions is relatively weak. Our results suggest that, unlike for calcium silicate minerals and clays, the forces acting between the weakly charged calcite surfaces are not significantly enhanced by Ca^2+^-induced attractive ion correlation effects.^62,66^ As such, the low adhesion between calcite surfaces measured in this work indicates that relatively high positive disjoining pressures act between calcite surfaces in water and in Ca^2+^-bearing solutions and that this repulsion is rather weakly affected by the changes in Ca^2+^ concentration.

The existence of nanometer-thin water films sustained on mineral grains by positive disjoining pressures has important implications for the geological and material deformation processes.^67^ As the thickness of these transport-enabling water films is sensitive to pore fluid composition, abrupt changes in chemical equilibrium and thus in type and concentration of ionic species can induce the spreading of reaction-driven fracturing fronts or trigger a sudden loss of cohesion within granular rocks or materials.

Reaction-driven fracturing can only continue provided that expanding mineral growth does not overcome the disjoining pressure between the mineral and the pore wall. Otherwise, the fluid film becomes too thin to allow the transport of species along the grain boundaries, and it can no longer support the mineral growth. Such a regulatory role of the positive disjoining pressure has been suggested in mineral replacement reactions by Zheng et al.^7^ The authors performed experiments where the thermodynamic driving force for mineral replacement was very high, but the reaction was found to shut down at much lower confining pressures than predicted thermodynamically. Although there are no suitable calculations or measurements of the disjoining pressure in this particular system, overcoming the disjoining pressure is a plausible explanation for this behavior. The extent to which the disjoining pressure can be modulated by changing the pore fluid composition is also of key importance in engineering applications, such as wellbore plugging with reactive cement^8^ or salt damage prevention in architectural objects.^9^ The microstructural evidence of closing fluid pathways well below the thermodynamic limit for crystallization pressure in such systems suggests that the solution- and mineral surface charge-sensitive disjoining pressure can determine whether a given porous reactive system will seal or fracture.

In this context, our results show that for relatively weakly charged minerals such as calcite, electrostatic double-layer forces can only play a limited role in maintaining positive disjoining pressures between mineral surfaces. On flat (104) calcite faces, the maximum height of the EDL barrier generally does not exceed several kPa in the absence of Ca^2+^. In natural systems, however, Ca^2+^ will always be present due to calcite dissolution and as only very low mM concentrations of potential-determining Ca^2+^ are needed to screen the surface charge of calcite, the EDL barrier will be even lower. In the presence of a significant crystallization pressure or overburden pressure in the subsurface, the EDL-related pressures for calcite appear insignificant. On the contrary, repulsive forces arising from calcite surface hydration^60^ and hydration of outer-sphere adsorbed Ca^2+^ ions^35^ are more likely to counteract the crystallization and overburden pressures and sustain nanometer-thin water films between contacting calcite surfaces.

We thus suggest that for ionic, weakly charged minerals such as calcite, ion-specific hydration effects will dominantly control the magnitude of the disjoining pressure acting between surfaces and thus the thickness of water films adsorbed within grain contacts. Less hydrated monovalent ions, such as sodium, which can adsorb closer to the calcite bulk termination,^26^ can decrease the thickness of water films, while more hydrated cations such as Ca^2+^ or Mg^2+^ at a given load will tend to keep the grains further apart. Interestingly, the very opposite effect is expected for minerals with a higher surface charge density, such as micas, with Ca^2+^ promoting strong attraction and Na^+^ causing repulsion between basal muscovite (001) surfaces.^48^

## 5 Conclusions

The mechanical behavior of rocks and granular materials is often controlled by grain-scale processes where the disjoining pressure acting across mineral surface-adsorbed water films is sensitive to the chemical composition of pore solutions. Sudden changes in chemical equilibria can affect the thickness of these water films and lead to the opening or closing of nanometer-thick fluid transport pathways between contacting grains, with potentially beneficial or adverse impacts on grain cohesion. In our study, we showed that the adhesive forces acting between the weakly charged calcite surfaces in Ca^2+^-bearing aqueous solutions are not strongly affected by varying Ca^2+^ concentrations. Compared to monovalent Na^+^, Ca^2+^ ions significantly reduce the adhesion measured between the two calcite surfaces and support thicker water films than Na^+^ at a given applied pressure and solution ionic strength. The repulsive electric double-layer forces cannot significantly weaken the adhesion in the presence of Ca^2+^, as the surface charge of calcite becomes quickly screened at very low mM concentrations of the potential-determining Ca^2+^ ions. As such, this loss in adhesion likely results from the repulsive hydration effects associated with the outer-sphere electrostatic binding of strongly hydrated Ca^2+^ cations. Our work points to the general importance of ion hydration properties in controlling the thickness of water films present between mineral grains with low surface charge density, where strongly hydrated ions can sustain and weakly hydrated ions tend to disrupt mineral surface-adsorbed hydration layers. As calcite-bearing rocks are important reservoirs affected by anthropogenic fluid extraction and storage operations, there is a further pressing need to understand the role of abundant inorganic ions on grain-scale forces and disjoining pressure acting between calcite surfaces.

## Supporting Information Available

Detailed parameters of all aqueous solutions used in this study, complementary ζ-potential modeling (PDF)

## Notes

Research Data associated with this article can be openly accessed in the Zenodo repository (CERN, OpenAIRE) at http://doi.org/10.5281/zenodo.4593071
