# Supplementary material for: Ca2+ Ions Decrease Adhesion between Two (104) Calcite Surfaces as Probed by Atomic Force Microscopy
Source: ACS Earth Space Chem. 2021 Oct 4;5(10):2827–38. doi: 10.1021/acsearthspacechem.1c00220 (PMC8543600; doi:10.1021/acsearthspacechem.1c00220)
Supplement: Supplementary file 1 — sp1c00220_si_001.pdf [file sp1c00220_si_001.pdf]

SUPPORTING INFORMATION FOR

Ca^{2+} ions decrease adhesion between two (104) calcite surfaces as probed by Atomic Force Microscopy

Joanna Dziadkowiec[a,*], Matea Ban[b], Shaghayegh Javadi[a], Bjørn Jamtveit[a], Anja Røyne[a]

[a]NJORD Centre, Department of Physics, University of Oslo, Oslo 0371, Norway

[b]Materials Testing Institute, University of Stuttgart, Pfaffenwaldring 2b, 70569 Stuttgart, Germany

*joanna.dziadkowiec@fys.uio.no

Table S1. Solution parameters for the low Ca^{2+} concentration calcite-saturated MilliQ water samples (type A solutions) used in the experiments E1 – E6. Only the initial and final pH values of the solutions are indicated; however, the pH was measured approximately every 5 minutes during the AFM experiments. The solutions marked with an asterisk were aged for the longer time (after a one day saturation process as described in the Methods section they were put aside, sealed with parafilm and later reused). The initial Ca^{2+} concentration was measured in solutions collected just before each experiment and the final Ca^{2+} concentration was measured in the solutions used in the AFM liquid cell.

**experiment**	**solution**	**injection order**	**measured pH**		**Ca^{2+} (mg/L)**	
			**pH initial**	**pH final**	**initial**	**final**
**E1**	pH 10	1	9,5	9,5	6,28	-
	pH 11	2	10,5	10,5	3,08	
	pH 12	3	11,8	-	3,02	
	pH 10	4	9,5	9,5	6,28	
	pH 11	5	-	-	3,08	
	pH 10	6	-	-	6,28	
**E2**	pH 10	1	9,0	8,8	7,94	-
	pH 11	2	10,3	9,9	2,90	2,82
	pH 12	3	11,8	11,8	2,80	2,65
	pH 10	4	9,1	8,8	7,54	7,22
	pH 11	5	10,3	10,0	2,60	3,12
**E3**	saturated pH 8.3	1	8,1	8,3	24,29	24,20
	saturated pH 8.3	2	8,2	8,2	24,28	24,25
	pH 10*	3	8,9	8,7	8,65	9,55
	pH 10	4	9,9	9,7	5,94	3,12
	pH 10	5	9,9	9,9	-	-
	pH 11*	6	10,3	10,1	2,57	2,93
	pH 11	7	10,8	10,6	4,23	4,36
	saturated pH 8.3	8	8,3	8,3	24,07	23,34

Table S1 continued:

experiment	solution	injection order	measured pH		Ca^{2+} (mg/L)	
			pH initial	pH final		final
**E4**	pH 10*	1	8,8	8,2	9,01	9,53
	pH 10	2	9,7	9,5	6,24	6,79
	pH 11*	3	10,2	9,9	2,69	0,05
	pH 11	4	10,8	10,5	4,28	3,68
	pH 12	5	11,8	11,8	2,64	2,80
	saturated pH 8.3	6	8,2	8,2	24,57	15,71
**E5**	saturated pH 8.3	1	8,1	8,2		23,78
	saturated pH 8.3	2	8,2	8,2		24,68
	pH 10*	3	8,4	8,3	13,45	15,12
	pH 10	4	9,6	9,4	6,49	7,46
	pH 11*	5	9,4	9,3	3,26	4,22
	pH 11	6	10,8	10,7	4,27	4,07
	pH 12	7	11,8	11,8	3,17	3,37
**E6**	saturated pH 8.3	1	8,2	8,2	25,45	-
	pH 10*	2	9,1	8,9	10,44	
	pH 10	3	9,5	9,3	6,71	
	pH 11*	4	9,2	9,1	4,18	
	pH 11	5	10,7	10,6	4,17	
	pH 12	6	11,8	11,8	3,06	

Table S2. Solution parameters for the high Ca^{2+} concentration calcite-saturated $CaCl_2$ type B solutions used in the experiments E7 – E14. Only the initial and final pH values of the solutions are indicated; however, the pH was measured approximately every 5 minutes during the AFM experiments.

experiment	solution	ionic strength (M)	injection order	measured pH	
				pH initial	pH final
E7	saturated pH 8.3	10^{-3}	1	8,23	8,27
	saturated pH 8.3	10^{-3}	2	8,22	8,23
	$CaCl_2$	0,25	3	7,28	7,33
	$CaCl_2$	0,5	4	7,17	7,19
	$CaCl_2$	1	5	6,98	7,01
E8	saturated pH 8.3	10^{-3}	1	8,24	8,29
	saturated pH 8.3	10^{-3}	2	8,22	8,21
	$CaCl_2$	0,25	3	7,24	7,29
	$CaCl_2$	0,5	4	7,18	7,21
	$CaCl_2$	1	5	6,99	7,04
E9	saturated pH 8.3	10^{-3}	1	8,23	8,30
	$CaCl_2$	0,25	2	7,30	7,32
	$CaCl_2$	0,5	3	7,17	7,22
	$CaCl_2$	1	4	7,00	6,99
E10	$CaCl_2$	0,5	1	7,03	7,10
	$CaCl_2$	1	2	6,97	7,02
	$CaCl_2$	2	3	6,79	6,98
E11	$CaCl_2$	0,5	1	7,07	7,00
	$CaCl_2$	1	2	6,90	6,94
	$CaCl_2$	2	3	6,71	6,78
E12	$CaCl_2$	0,5	3	7,18	7,16
	$CaCl_2$	1	2	7,01	6,98
	$CaCl_2$	2	1	6,72	6,76
E13	$CaCl_2$	0,5	3	7,24	7,18
	$CaCl_2$	1	1	6,97	7,06
	$CaCl_2$	2	2	6,79	6,86
E14	saturated pH 8.3	10^{-3}	4	8,27	8,21
	$CaCl_2$	0,5	3	7,22	7,16
	$CaCl_2$	1	2	6,98	6,98
	$CaCl_2$	2	1	6,67	6,74

Table S3. Parameters of the low Ca^{2+} concentration, calcite-saturated solutions used in the streaming zeta potential measurements.

solution	injection order	measured pH		Ca^{2+} (mg/L)	conductivity (mS/m)
		pH initial	pH final		
pH 10	1	8,27	8,07	7,28	6,4
pH 11	2	9,69	9,42	1,36	10,7
pH 12	3	11,05	10,90	1,12	209,6
saturated pH 8.3	4	8,2	9,0	19,69	15,2

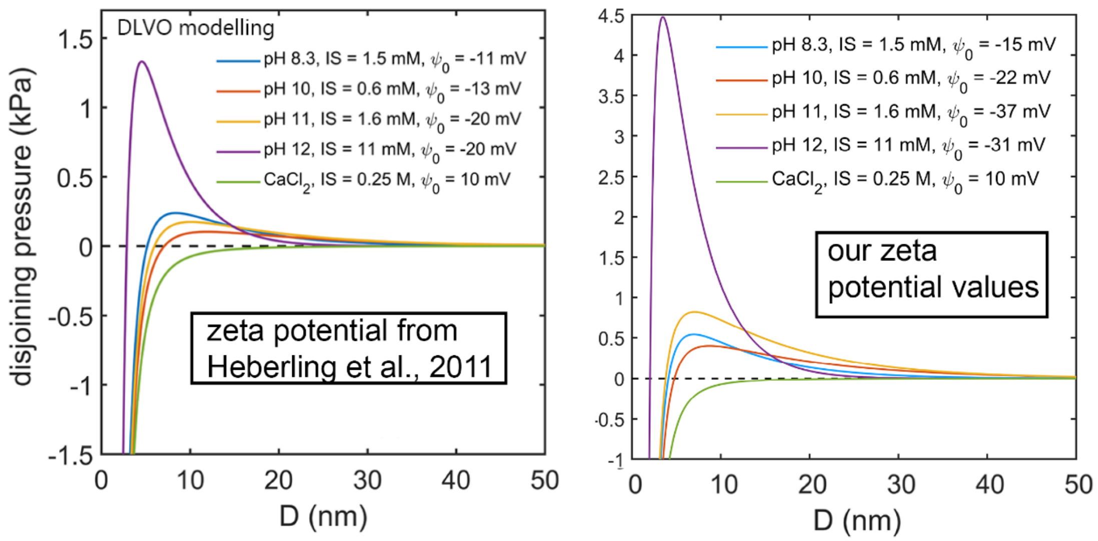

Figure S1. Modelling of the DLVO disjoining pressure as a function of separation (D) between two flat calcite surfaces in low concentration solutions with pH between 8.3 and 12 and lowest IS=0.25 M $CaCl_2/CaCO_3$ solution (see Eqs. 2 – 4 in the manuscript). The left panel is reproduced from Figure 4 in the manuscript with indicated surface potential values adapted from Heberling et al. (2011)[1]. The right panel shows the DLVO modelling using zeta potential values measured in our study as described in the manuscript (apart from the 0.25 M $CaCl_2/CaCO_3$ where we used the same zeta potential values as in the left panel). In both cases, the Hamaker constant for two calcite surfaces across the water $A_{cwc} = 1.44 \cdot 10^{-20}$ J was adapted from Bergström (1997)[2]. The IS strength of the solutions was calculated using PhreeQc geochemical modeling software[3] and verified across the measured pH and Ca^{2+} concentrations. Note differences in y-axes scale.

References:

1. Heberling, F.; Trainor, T. P.; Lützenkirchen, J.; Eng, P.; Denecke, M. A.; Bosbach, D., Structure and reactivity of the calcite–water interface. *Journal of colloid and interface science* **2011,** *354* (2), 843-857.
2. Bergström, L., Hamaker constants of inorganic materials. *Advances in colloid and interface science* **1997,** *70*, 125-169.
3. Parkhurst, D. L.; Appelo, C. *Description of input and examples for PHREEQC version 3: a computer program for speciation, batch-reaction, one-dimensional transport, and inverse geochemical calculations*; 2328-7055; US Geological Survey: 2013.
